# Supplementary material for: Information-based TMS to mid-lateral prefrontal cortex disrupts action goals during emotional processing
Source: Nat Commun. 2024 May 20;15:4294. doi: 10.1038/s41467-024-48015-8 (PMC11106324; doi:10.1038/s41467-024-48015-8)
Supplement: Supplementary file 1 — Supplementary Information [file 41467_2024_48015_MOESM1_ESM.pdf]

Supplementary Information

Supplementary Methods

AGNG task: test-retest reliability

Because our experiment required modifying and adapting typically fully-blocked AGNG task designs into a mixed block/event-related task, we estimated the test-retest reliability across the different fMRI sessions we conducted. For context, Go/No-Go tasks have typically moderate-to-high test-retest reliability. Previously reported test-retest Pearson's *r*s for similarly designed paradigms have ranged between *r*s = [.62 -.76] for commission errors (i.e., false alarms in No-Go trials; Bender et al. 2016: *r* = .62; Kertzman et al. 2008: *r* = .64; Weafer et al. 2013: *r* = .65; Hedge et al. 2017: ICC = .76[1–4]; reviewed in[5]). For completeness, we note that test-retest reliability of RTs in Go/No-Go tasks (albeit not changed by TMS in our task) have included ICCs= 0.63-0.74[4] and *r* = .88[1–5].

First, we examined the test-retest reliability of the task as estimated using the behavior obtained in the two TMS+fMRI sessions, which were conducted on average 5.6 days apart (*SD* = 8.2 days). For commission errors, we found comparable task reliability to what had been previously reported in the literature, ranging from [*r*s = 65 – .68]: (TMS Session 1 vs 2 *Collapsed across emotional valences*: *r* = 0.689, *p* = 1.8e-05, 95% CI = [0.443, 0.839]; *Negative valence*: *r* = 0.651, *p* = 7.3e-05, 95% CI = [0.386, 0.817]; *Positive valence*: *r* = 0.688, *p* = 1.9e-05, 95% CI = [0.442, 0.838]). When examining the test-retest reliability between the baseline (no TMS) vs. Control (S1) fMRI sessions, which were conducted on average 98 days apart (*SD* = 43 days), reliability was moderate, ranging from [*r*s = 0.55–0.66]: (Baseline vs. Control TMS session: *Collapsed across emotional valences:* *r* = 0.661, *p* = 5.2e-05, 95% CI = [0.4, 0.822]; *Negative valence:* *r* = 0.596, *p* = 4e-04, 95% CI = [0.306 0.785]; *Positive valence*: *r* = 0.553, *p* = 0.0013, 95% CI = [0.247 0.758]).

Test-retest reliability of AGNG RT data were as follows: TMS Session 1 vs 2 *Collapsed across emotional valences* (*r* = 0.912, *p* = 9e-13, 95% CI = [0.825, 0.957; *Negative valence*: *r* = 0.927, *p* = 6.9e-14, 95% CI = [0.853, 0.965]; *Positive valence:* *r* = 0.876, *p* = 1.1e-10, 95% CI = [0.756, 0.939]. Test-retest reliability for RTs between baseline (no TMS) and Control (S1) TMS sessions were comparable: (*Collapsed across emotional valences:* *r* = 0.818, *p* = 2e-08, 95% CI = [0.652, 0.909]; *Positive only:* *r* = 0.803, *p* = 5.6e-08, 95% CI = [0.627, 0.901]; *Negative only:* *r* = 0.823, *p* = 1.4e-08, 95% CI = [0.661, 0.911]).

Supplementary Results

MVPA Classifier Results

mid-LPFC action goal decoding: Individual-level results

Examination of individual-level data indicated that 22/31 (71%) subjects showed action-goal classifier performance (AUC) > 0.5 in the baseline fMRI session (10/31 significantly above chance at the single subject/session level) and 22/31 (71%) showed action-goal AUC > 0.5 in the Control TMS fMRI session (15/31 were statistically above chance at the single subject/session level) in an unbiased anatomical mid-LPFC ROI (**Figure S1 A-B**).

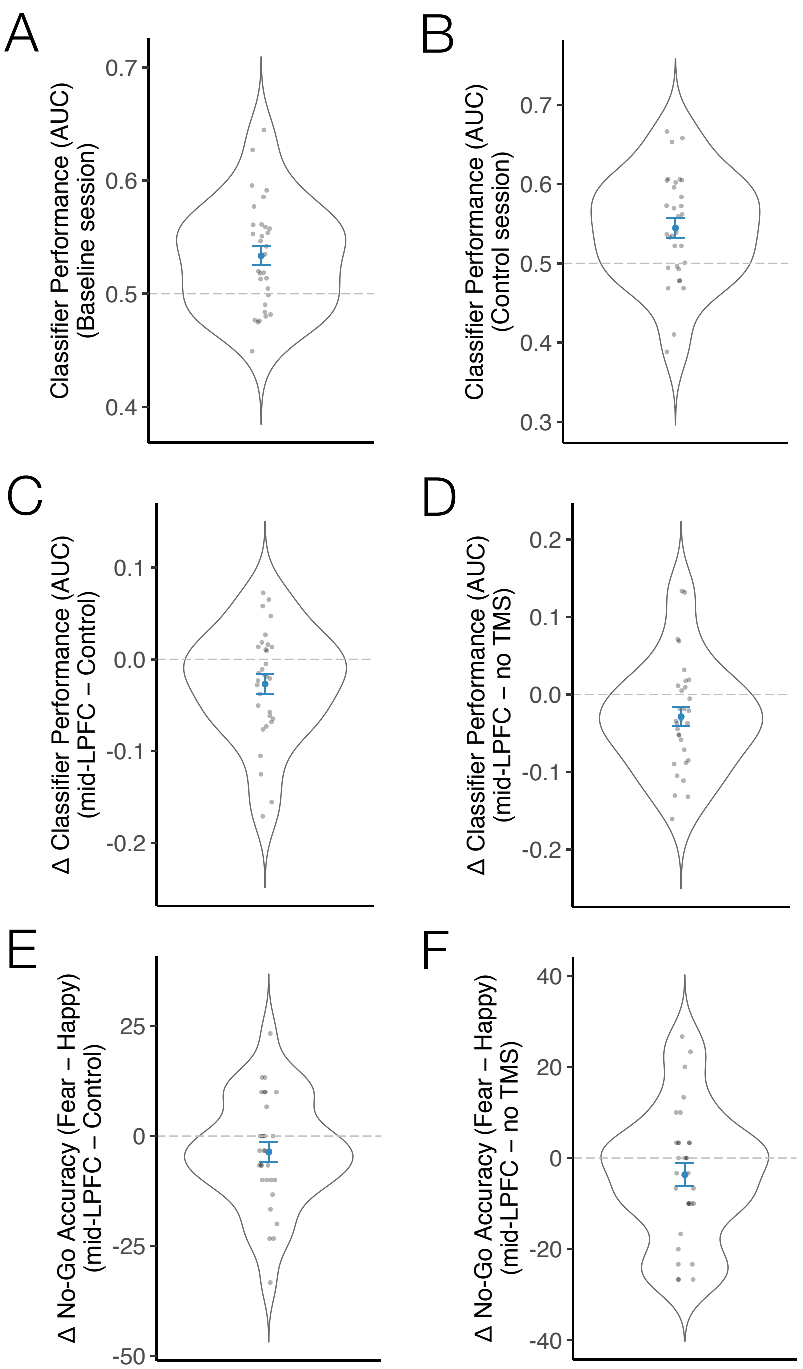

Figure S1. (**A & B)** Subject-level classifier performance (AUC) for action-goal decoding (Go/No-Go) in the anatomically defined mid-LPFC ROI observed in **(A)** the baseline (no TMS) fMRI session and in **(B)** the TMS Control (S1) fMRI session. **(C & D)** Subject-level change scores in classifier accuracy (AUC) for action goal decoding in individualized mid-LPFC ROIs following mid-LPFC cTBS are shown relative to **(C)** the TMS Control (S1) fMRI session and **(D)** the baseline (no TMS) fMRI session. **(E & F)** Subject-level change scores observed in No-Go accuracy (Negative – Positive) in the AGNG task following mid-LPFC cTBS are shown relative to **(E)** the TMS Control (S1) session and **(F)** the baseline (no TMS) session. The data of all participants (N = 31) are plotted (each grey dot represents a subject). Blue: mean values & error bars (±1 SEM).

Non-parametric analysis

As detailed in the Methods and in the main text, cTBS to mid-LPFC reduced the strength of action-goal decoding (Go vs. No-Go AUC) in individualized mid-LPFC sites compared to both baseline (no TMS) and Control (S1) TMS sessions as indicated by the performance of a logistic classifier using a leave-one-run-out cross validation approach, which was combined across subjects and runs using a mixed model (all *p*s < 0.0023; see **Figure 2A** and main text for details).

To examine the robustness of these results to possible violations of normality, we also analyzed the data using a non-parametric method. Specifically, we obtained an empirically derived null distribution against which we compared classifier decoding results by running shuffling class labels (n=500 permutations) and computing Go vs. No-Go classifier accuracy of randomly shuffled data. We examined whether classifier performance was significantly different from chance by comparing it to the empirically derived null distribution. This non-parametric approach corroborated our key findings, as follows: above-chance action-goal decoding in mid-LPFC was evident in both baseline (no TMS) and Control (S1) sessions ($Z = 3.706$, $p = 0.0002$ and $Z = 3.124$, $p = 0.002$, respectively), but was at chance following cTBS to LPFC, ($Z = 0.085$, $p = 0.453$) (**Figure S2**).

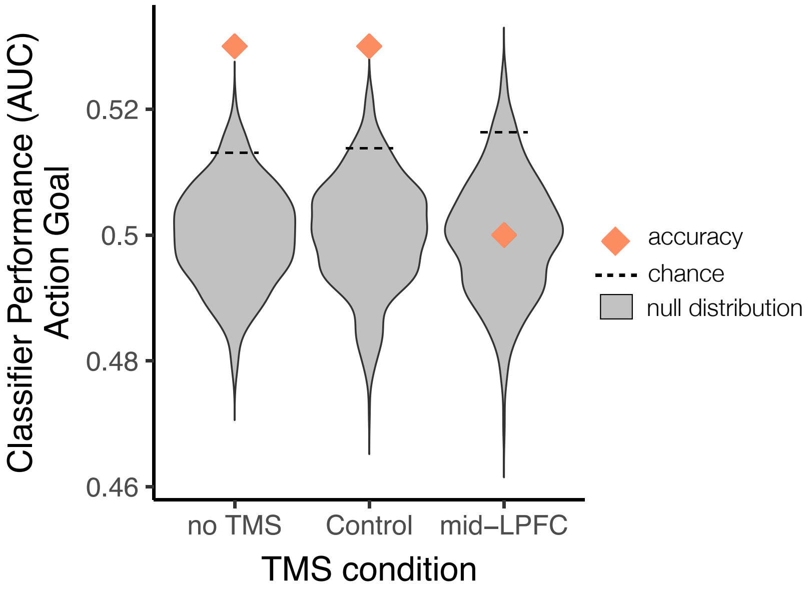

Figure S2. Multivariate classifier performance (decoding of action goal: Go vs. No-Go) from individualized mid-LPFC sites is plotted (AUC) relative to an empirically derived null distribution as a function of TMS condition. Following cTBS to mid-LPFC, action goals were no longer decodable above chance from mid-LPFC neural activity patterns, which contrasts to above-chance decoding observed in both baseline (no TMS) and Control (S1) sessions. The data of all participants (N = 31) are plotted. Orange: average accuracy. Gray: permuted null distribution (n = 500 permutations). Dotted line: chance level based on the empirically derived null distribution.

Behavioral Results

Mood

TMS site administration (mid-LPFC vs. S1) did not differentially impact self-reported positive mood (PANAS Now PA: TMS: $t(27) = -0.748$, $p = 0.461$) or negative mood (PANAS Now NA: TMS: $t(27) = -0.61$, $p = 0.547$). In addition, across individuals, the magnitude of TMS-evoked changes in subjective negative and positive mood were unrelated to changes in task behavior, all *p*s > 0.31: ΔS1-LPFC Negative Affect and ΔS1–LPFC No-Go Accuracy (Fear – Happy) $r = 0.009$, $p = 0.96$; Δ S1–LPFC Positive Affect and ΔS1–LPFC No-Go Accuracy (Fear – Happy) $r = 0.063$, $p = 0.76$; ΔS1–LPFC Negative Affect and ΔS1–LPFC RT (Fear – Happy) $r = -0.003$, $p = 0.99$; ΔS1–LPFC Positive Affect and ΔS1–LPFC RT (Fear – Happy) $r = -0.206$, $p = 0.31$.

Response Time

In the AGNG task, Response Times (RT) in "Go" trials are typically shorter in response to positively valenced "Go" targets, putatively reflecting a goal-congruent (i.e., approach) facilitatory impact of appetitive states induced by positive stimuli, such as happy faces[6–16]. We replicated this effect as indicated by the highly robust (and consistent across sessions and TMS sites) impact of emotional valence on RT in correct "Go trials" (Emotional valence main effect: $F = 56.12$, $p < 0.001$), which was unaffected by TMS (**Figure S3**) (Emotional valence * cTBS site interaction $F = 0.0376$, $p > 0.963$). Of note, RTs were slightly slower following cTBS to the Control (S1) site compared to the initial baseline (no TMS) session ($p = 0.0164$), but, importantly, RTs obtained in the two TMS+fMRI sessions did not differ (LPFC vs. Control (S1) $p = 0.175$). RTs following cTBS to LPFC were also comparable to RTs observed in the baseline (no TMS) session ($p = 0.236$). In summary, RT data in the current sample replicate well-known findings of facilitation by positive emotional valence, but were unaffected by LPFC intactness. Collectively, these data underscore results previously discussed in the main text, which indicate that cTBS to LPFC impaired performance specifically in "No-Go" (rather than in "Go") trials.

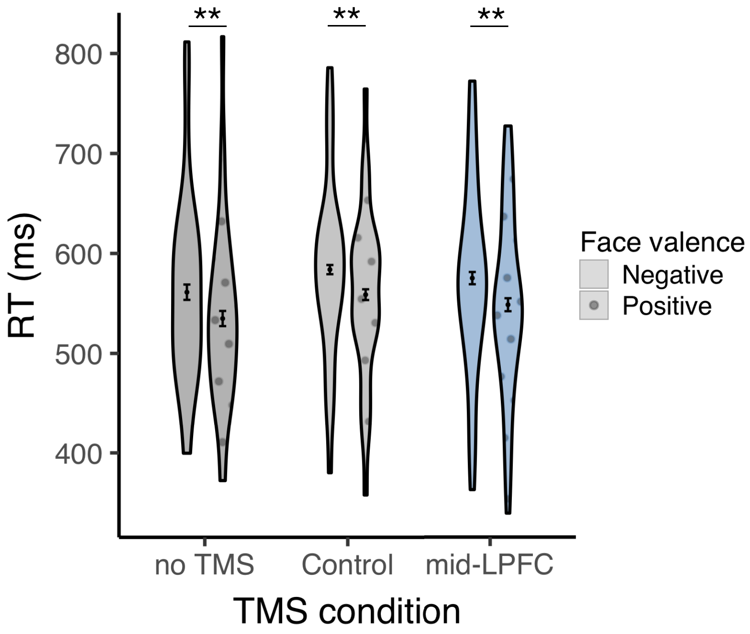

Figure S3. Response time (RT) data are plotted as a function of TMS condition and emotional valence of facial expressions (Negative vs. Positive). The data of all participants (N = 31) are plotted. Colors denote TMS condition: Dark gray = no TMS; Light gray = Control (S1) TMS; Blue = mid-LPFC TMS. Texture denotes emotional valence condition: Plain = Negative; Circles = Positive. Data are presented as mean values per condition. Error bars: ±1 SEM of the within-subjects difference between conditions[17].

Accuracy

As detailed in the Results section, fearful faces increased accuracy in "No-Go" trials relative to happy faces in both baseline (no TMS) and Control (S1) TMS sessions (*p*s < 0.0005)—but not after cTBS was administered to mid-LPFC ($p = 0.597$; cTBS site*action goal*valence interaction $F = 4.133$, $p = 0.016$)—suggesting that cTBS to mid-LPFC abolished the usual facilitatory effect of emotional valence on avoidance-congruent goals in the AGNG task (see **Figure 2B** and main text for details). The modulatory impact of cTBS on No-Go Accuracy was expressed primarily in trials where the No-Go target was a fearful face: performance on fearful-face No-Go trials was lower following cTBS to mid-LPFC compared to both baseline and TMS Control (S1) sessions ($p < 0.0001$ and $p = 0.0022$, respectively); performance in fearful-face trials did not differ between baseline and TMS Control (S1) sessions ($p > 0.129$). In contrast, when No-Go targets were a happy face, No-Go accuracy following cTBS to LPFC vs Control (S1) did not significantly differ from each other ($p > 0.521$), and performance at baseline was equivalent to performance in the Control (S1) session ($p = 0.13$), although baseline accuracy was higher compared to after cTBS to LPFC ($p = 0.024$). Neutral face No-Go accuracy was not modulated by cTBS (*p*s > .3).

Performance in "Go" trials was unaffected by emotional valence (happy vs. fear: no TMS $p > 0.38$; Control (S1) $p > 0.74$; mid-LPFC $p > 0.47$; see **Figure S4** for all conditions plotted as a function of cTBS site, action goal, and emotional valence). Moreover, performance in "Go" trials (collapsed across valences) did not differ when comparing mid-LPFC vs. Control (S1) TMS conditions. Performance in both TMS sessions was lower relative to baseline (no TMS) (*p*s < 0.01), thereby likely reflecting non-specific TMS and/or fatigue effects from the longer TMS+fMRI (vs. fMRI only) sessions.

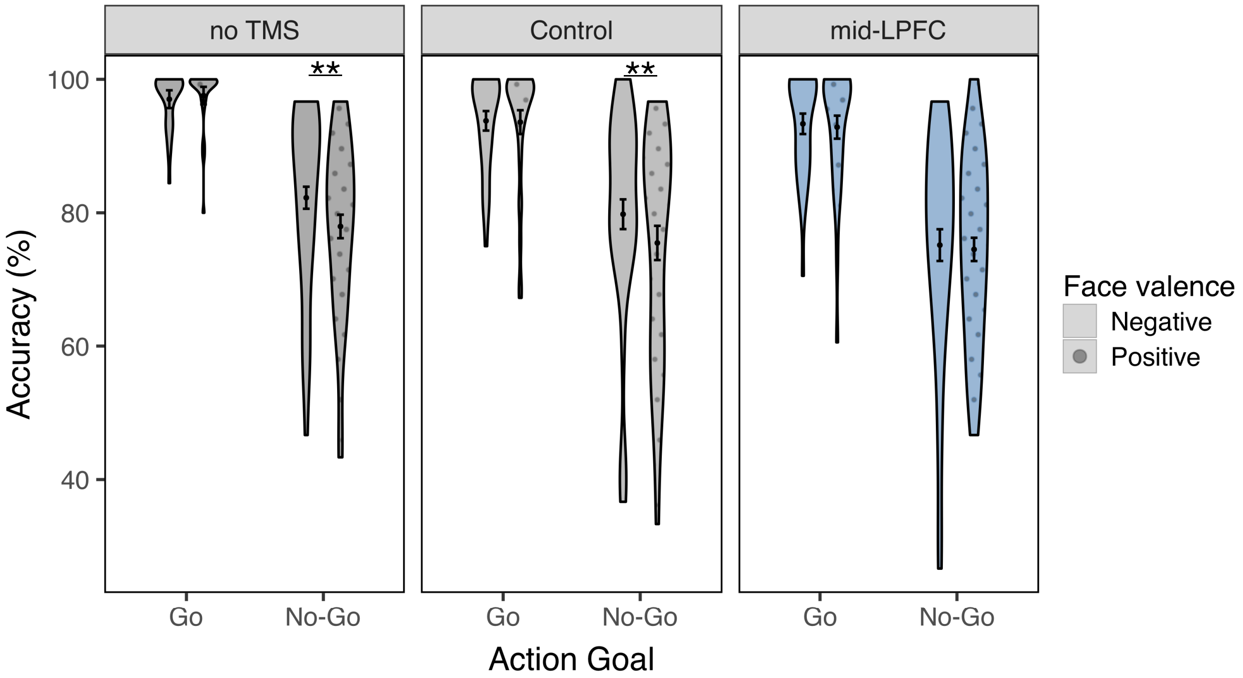

Figure S4. Task accuracy data are shown as a function of TMS site, action goal (Go vs. No-Go) and emotional valence of facial expressions (Negative vs. Positive). The data of all participants (N = 31) are plotted. Colors denote TMS condition: Dark gray = no TMS; Light gray = Control (S1) TMS; Blue = mid-LPFC TMS. Texture denotes emotional valence condition: Plain = Negative; Circles = Positive. Data are presented as mean values per condition. Error bars: ±1 SEM of the within-subjects difference between conditions[17].

PPI Results

Whole-brain analysis

A psychophysiological interaction analysis (PPI) of negative emotional processing during the AGNG task following mid-LPFC cTBS (vs. S1) revealed reduced functional coupling between mid-LPFC and a set of regions including mPFC-frontopolar cortex, precuneus as well as visual cortical sites (detailed in the main text; **Figure 3A**) (whole-brain cluster-level corrected for multiple comparisons at $Z < -3.1$, $p < 0.05$). Collectively, these regions overlapped primarily with the DMN and visual cortical networks as defined by Yeo et al.[18] (46.58% of whole-brain, cluster-corrected voxels fell within the DMN and 34% in visual network (3.21% of the DMN and 3.24% of Yeo-7 visual-cortical networks overlapped with this cluster); when examining the FP/mPFC cluster, we found that 57.8% of corrected voxels overlapped with the DMN vs. 13.295% with the ventral-attention network; for additional details, see **Tables S2** and **S3**).

PPI & MVPA Mixed-effects model

In alignment with results reported in the main manuscript (**Figure 3B**), emotion-dependent functional coupling of mid-LPFC sites and frontopolar (FP) cortex (defined using an a-priori anatomical FP ROI) was associated with stronger action-goal classifier evidence (Go vs. No-Go AUC) in mid-LPFC, suggesting that frontopolar function may facilitate conveying of affective signals that inform action goals represented in LPFC (across-runs mixed-model $B = 0.01$, $SE = 0.004$, $t = 2.57$, $p = 0.011$).

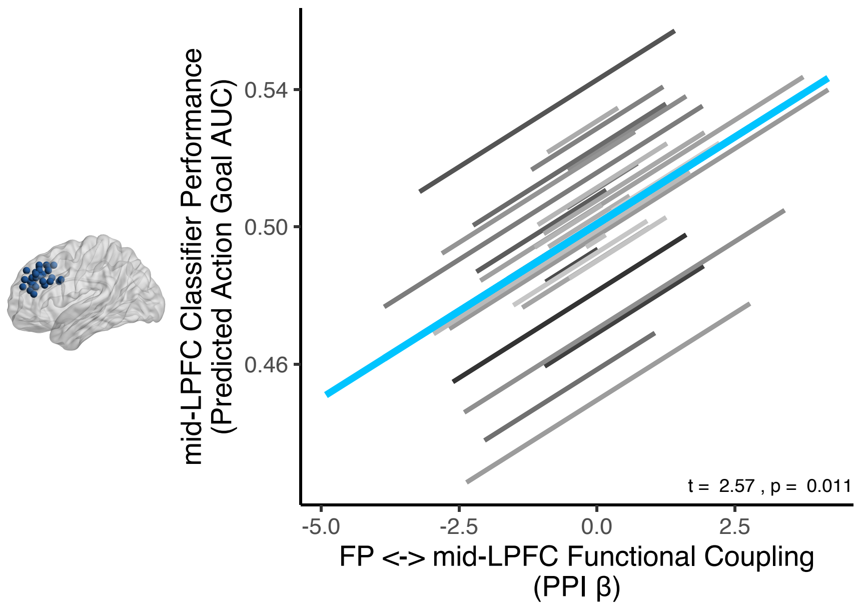

Figure S5. Greater frontopolar—mid-LPFC coupling (PPI Beta) during negative (vs. positive) emotional processing is associated with greater mid-LPFC action-goal decoding (Go vs. No-Go AUC) in the AGNG task. The data of all participants (N = 31) are plotted.

Control Analyses

Probing representational specificity: Action-goal vs. emotional-valence decoding changes after mid-LPFC cTBS

Emotional valence (Positive vs. Negative faces) was not decodable above chance from mid-LPFC voxels at baseline (AUC M = 0.502, B = 0.002, SE = 0.011, t = 0.178, Cohen's d = 0.028, p = 0.86). Following cTBS to mid-LPFC, decoding of emotional valence remained at chance in this region (AUC M = 0.498 SE = 0.00869, t = -0.241, Cohen's d = 0.052, p = 0.811), with no change as a function of TMS site (cTBS * region interaction: F = 1.419, p = 0.243; pairwise comparisons: mid-LPFC vs. Control (S1) t = -1.163, Cohen's d = 0.11, p = 0.246; mid-LPFC vs. baseline t = 0.434, Cohen's d = 0.068, p = 0.664) (**Figure S6**). Classifier performance after Control (S1) TMS remained non-significantly different from chance (t = -1.295, Cohen's d = 0.233, p = 0.205) and did not differ from baseline (t = 1.634, Cohen's d = 0.18, p = 0.103).

Further demonstrating the informational specificity of our cTBS approach targeting mid-LPFC action-goals, the cTBS-driven reduction in classifier evidence for *action goals* in mid-LPFC (**Figure 2**) was significantly stronger than for *emotional valence*, as indicated by a significant cTBS*information type (action vs. valence) interaction in mid-LPFC voxels (F = 4.207, p = 0.015).

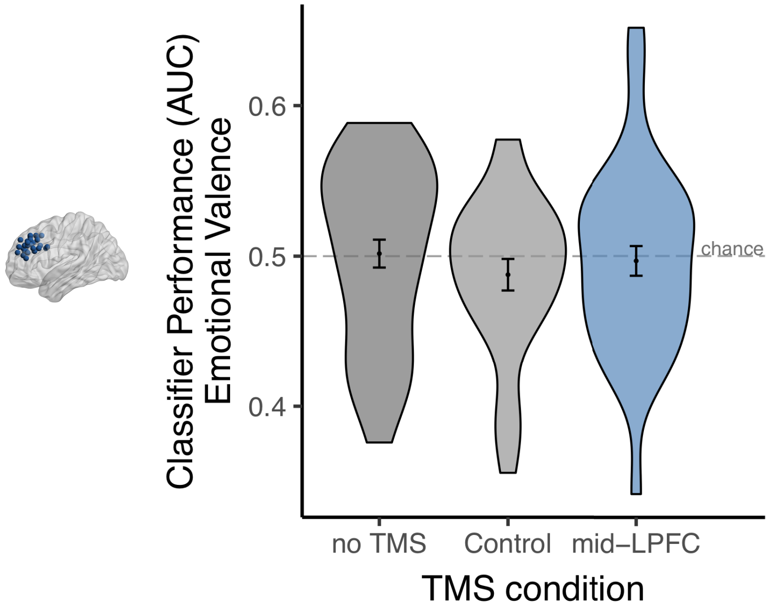

Figure S6. Multivariate classifier performance for decoding of emotional valence (Positive vs. Negative) in subject-specific mid-LPFC TMS sites is plotted (AUC) as a function of TMS condition. In contrast to action-goal evidence, classifier performance was at chance for emotional valence decoding at baseline, and unchanged by cTBS to mid-LPFC. The data of all participants (N = 31) are plotted. Colors denote TMS condition: Dark gray = no TMS; Light gray = Control (S1) TMS; Blue = mid-LPFC TMS. Data are presented as mean values per condition. Error bars: ±1 SEM of the within-subjects difference between conditions[17].

Univariate vs. multivariate impact of mid-LPFC cTBS

cTBS to mid-LPFC altered mid-LPFC multivariate classifier evidence of action goals without changing univariate activity (cTBS site main effect n.s. $F = 0.92$, $p = 0.409$). The interaction of action goal (Go vs. No-Go) * cTBS site on mid-LPFC univariate activity was also non-significant ($F = 0.69$, $p = 0.506$) (**Figure S7**), thereby underscoring the relative functional specificity of the impact of information-guided TMS on multivariate (vs. univariate) action-goal signals.

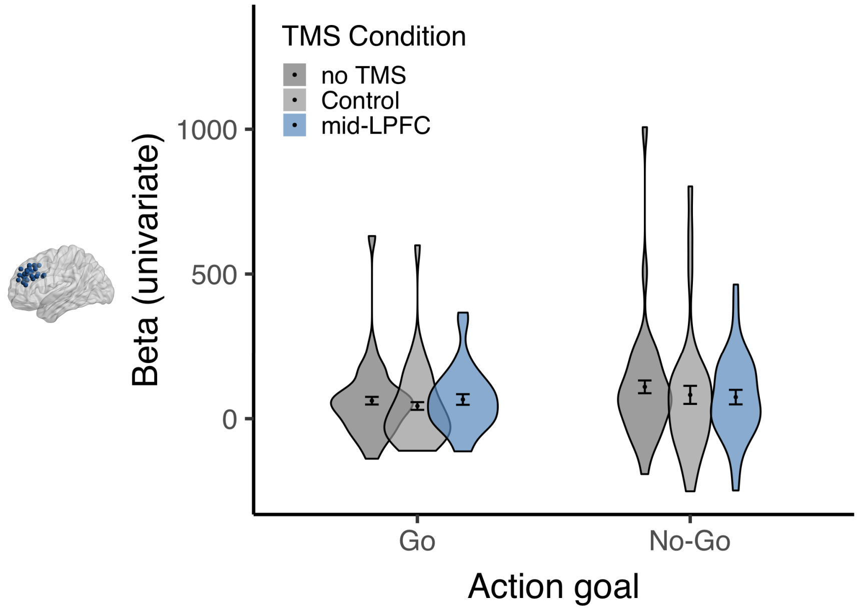

Figure S7. Univariate activity is plotted as a function of Action Goal (Go vs. No-Go) and TMS Condition (no TMS, Control/S1, and mid-LPFC). Information-guided cTBS to mid-LPFC reduced multivariate decoding of action goals (Go vs. No-Go; **Figure 2A**) without altering overall univariate activity in mid-LPFC. The data of all participants (N = 31) are plotted. Colors denote TMS condition: Dark gray = no TMS; Light gray = Control (S1) TMS; Blue = mid-LPFC TMS. Data are presented as mean values per condition. Error bars: ±1 SEM of the within-subjects difference between conditions[17].

Regional specificity of MVPA changes by mid-LPFC cTBS: anatomical vs. individualized mid-LPFC ROIs

In the current study, we targeted mid-LPFC based on the location of individualized action-goal (Go vs. No-Go) multivariate representations. We found that the strength of action-goal decoding in individualized mid-LPFC sites was reduced following cTBS to mid-LPFC (compared to baseline and Control (S1) sessions, *p*s < 0.0023; see main text and **Figure 2A** for details).

To examine whether this effect was regionally specific, we tested whether cTBS to mid-LPFC reduced action-goal representations in an anatomically defined (as opposed to functionally-defined and individualized) mid-LPFC ROI. We found that cTBS to mid-LPFC did *not* change the strength of action-goal representations when an anatomically defined mid-LPFC mask was examined: in this anatomical ROI, action-goal decoding remained significant following cTBS to mid-LPFC ($M = 0.545$, $B = 0.045$ ($SE = 0.01$), t = 4.47, Cohen's d = 0.764, $p < 0.001$) and was comparable to decoding observed in both baseline (no TMS) and TMS Control (S1) sessions ($M = 0.533$, $B = 0.033$ ($SE = 0.01$), t = 3.215, Cohen's d = 0.707, $p = 0.007$ and $M = 0.545$, $B = 0.045$ ($SE = 0.012$), t = 3.874, Cohen's d = 0.655, $p = 0.001$, respectively; main effect of cTBS site was n.s. at $p > 0.173$) (**Figure S8**), which contrasts starkly with results obtained when examining functionally-specific, individualized cTBS sites that were targeted (**Figure 2A** and **Figure S2**).

Accordingly, the interaction of ROI * TMS condition (ROI: anatomical vs. individualized (functional) * TMS condition: baseline, Control vs. mid-LPFC) was significant, (F = 5.733, $p = 0.003$), indicating that the change in action-goal decoding in mid-LPFC by cTBS was specific to individualized (*vs. anatomically defined*) mid-LPFC targets. Namely, action-goal decoding in mid-LPFC was significantly disrupted by cTBS only in individualized, functionally defined ROIs, but not in the larger, anatomical mid-LPFC ROI (mid-LPFC AUC pairwise ROI comparison: $t = 4.466$, $p < 0.0001$).

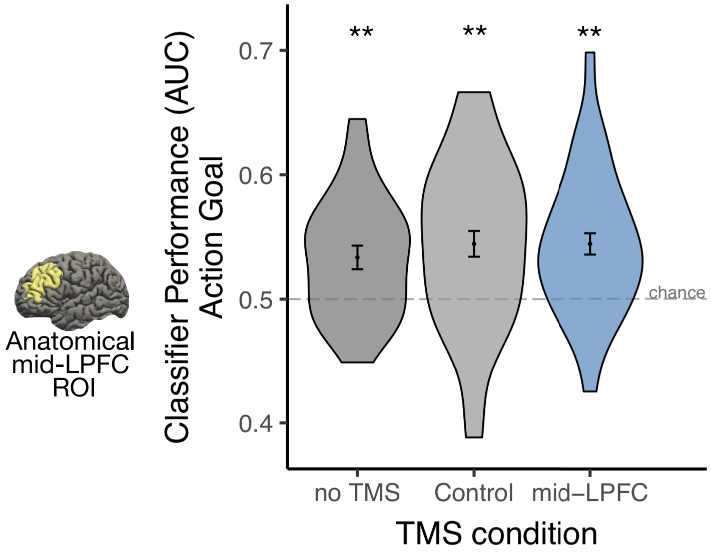

Figure S8. Multivariate classifier performance (decoding of action goal: Go vs. No-Go) from an anatomically defined mid-LPFC region is plotted (AUC) as a function of TMS condition. Following

cTBS to mid-LPFC, action-goal remained decodable above chance from an anatomically defined mid-LPFC mask, in stark contrast with results observed when probing individual-specific and functionally defined mid-LPFC sites (**Figure 2A**). The data of all participants (N = 31) are plotted. Colors denote TMS condition: Dark gray = no TMS; Light gray = Control (S1) TMS; Blue = mid-LPFC TMS. Data are presented as mean values per condition. Error bars: ±1 SEM of the within-subjects difference between conditions[17]. ** $p < 0.01$

Regional specificity of MVPA changes by mid-LPFC cTBS: individualized vs. non-individualized (but functionally defined) mid-LPFC ROIs

In an additional analysis probing the regional specificity of the reported results, we next examined whether action-goal decoding in individualized mid-LPFC ROIs—and their change by mid-LPFC TMS—was stronger than in *non-individualized*, but equivalently-sized and functionally-defined mid-LPFC ROIs obtained from other subjects. To do so, for each subject, we extracted and averaged action-goal classifier AUC by TMS condition from *all other subjects'* mid-LPFC ROIs that did not spatially overlap with that subject's own individualized mid-LPFC ROI. This analysis permitted addressing (A) whether subject-specific mid-LPFC ROIs are stable (if so, action-goal decoding should be stronger in individualized (compared to non-individualized) mid-LPFC sites in an independent session, here, the Control/S1 session); and (B) whether the reduction in mid-LPFC action-goal decoding by mid-LPFC cTBS was more pronounced in the targeted, individualized (compared to non-individualized) mid-LPFC ROIs.

As shown in **Figure S9**, action-goal decoding in mid-LPFC obtained in an independent session—the Control/S1 session—was stronger in individualized ROIs compared to non-individualized mid- ROIs (p = 0.045). This indicates that individualized (information-based) ROIs were stable and better captured action-goal information in mid-LPFC (compared to non-individualized mid-LPFC ROIs). Second, whether mid-LPFC ROIs were individualized significantly modulated the main effect of TMS condition, as indicated by a ROI * TMS condition interaction, F = 9.226, p = 0.00011. Specifically, the reduction of classifier action-goal decoding in mid-LPFC by cTBS was specific to individualized (targeted) mid-LPFC ROIs compared to in non-individualized ROI. In contrast to results obtained in subject-specific (individualized) ROIs (see manuscript for details), the main effect of TMS condition was <u>absent</u> when examining equally sized, but non-individualized mid-LPFC ROIs (TMS main effect F = 1.617, p > 0.214). Action-goal decoding in non-individualized (non-targeted) mid-LPFC ROIs remained above chance following mid-LPFC cTBS ($p_{vs.chance}$= 0.004) and was <u>not</u> reduced compared to either baseline (no TMS) (p > 0.9405) or Control (S1) TMS sessions (p > 0.398). Action goal decoding following mid-LPFC (putatively inhibitory) cTBS was significantly *lower* in individualized mid-LPFC ROIs targeted by TMS compared to non-individualized mid-LPFC ROIs (p = 0.039) (**Figure S9**).

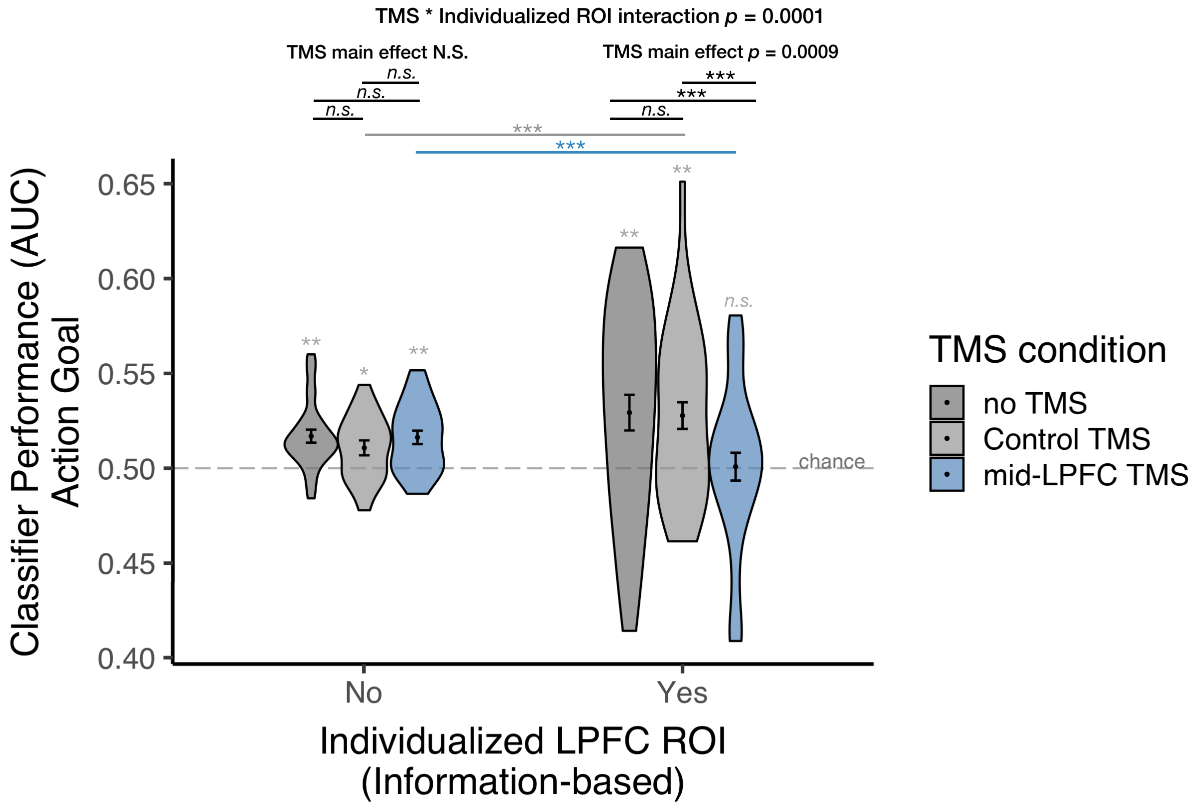

Figure S9. **Multivariate classifier performance in LPFC as a function of whether the LPFC ROI examined was individualized (information-based) versus not.** Multivariate classifier performance of action-goal decoding (Go vs. No-Go classifier AUC) from mid-LPFC sites is plotted as a function of TMS condition and whether the location of the LPFC ROI examined was individualized based on subject's location of peak classifier performance at the baseline fMRI session (no TMS) (analyses reported in the main manuscript; Individualized ROI: 'Yes') versus from *other subjects'* LPFC ROIs (mean AUC) (Individualized ROI: 'No'). Whether the mid-LPFC ROI examined was individualized significantly modulated the main effect of TMS condition, as indicated by a significant ROI * TMS condition interaction, $F = 9.226$, $p = 0.00011$. As reported in the manuscript, following cTBS to mid-LPFC, action-goal decoding (AUC) in subject's individualized (and targeted by TMS) mid-LPFC ROIs dropped to chance levels ($p_{vs.chance} > 0.885$), and was significantly reduced compared to <u>both</u> baseline (no TMS) (p = 0.0006) and Control (S1) TMS sessions (p = 0.0007), TMS main effect F = 7.117, p = 0.0009. Notably, a TMS condition main effect is absent when examining equally sized, but non-individualized LPFC ROIs (i.e., average Go vs. No-Go classifier AUC obtained from other individuals), TMS main effect *N.S.* ($F = 1.617$, $p > 0.214$). Specifically, decoding in non-individualized mid-LPFC ROIs remained above chance ($p_{vs.chance}= 0.004$) and was <u>not</u> reduced following mid-LPFC cTBS compared to either baseline (no TMS) ($p > 0.9405$) or Control (S1) TMS sessions ($p > 0.398$). Critically, TMS significantly reduced classifier decoding in individualized (targeted) mid-LPFC ROIs compared to in non-individualized ROI ($p = 0.039$). Moreover, as would be expected if individualized (information-based) ROIs captured action-goal information in mid-LPFC better and more reliably than non-individualized ROIs, action-goal decoding in mid-LPFC obtained in an independent session—the Control/S1 session—was stronger in individualized compared to non-individualized ROIs ($p = 0.045$). The data of all participants (N = 31) are plotted. Colors denote TMS condition: Dark gray = no TMS; Light gray = Control (S1) TMS; Blue = mid-LPFC TMS. Data are presented

as mean values per condition. Error bars: ±1 SEM of the within-subjects difference between conditions[17].

*<u>P values legend:</u> ***p ≤ 0.001; ** p <.01; * p < .05*
Light gray: individual condition p values (action goal decoding) against chance
Medium gray: pairwise p value for individualized mid-LPFC ROI in the Control TMS condition
Blue: pairwise p value for individualized mid-LPFC ROI in the mid-LPFC TMS condition
Black: pairwise p values for TMS condition contrasts (per ROI condition)

TMS sites: scalp-cortex distances

Following an anonymous Reviewer suggestion, and in light of the fact that distances from scalp to cortex can vary across individuals and TMS targets, we estimated scalp-to-cortex distances for mid-LPFC and S1 TMS sites to assess whether this may account for inter-subject variability of the influence of TMS. To do so, we calculated the approximate difference in the effective dose of TMS per subject and TMS site using the Stokes et al. (2005) equation[19] and examined associations between the estimated distance-adjusted TMS intensity (per site) and the neural and behavioral effects reported in this study.

The Euclidean distances between scalp coordinates and TMS cortical locations for the available TMS projection data (N = 29/31 subjects) for mid-LPFC and the Control site (S1) were as follows: mid-LPFC $M_{distance}$ = 22.133 mm (*SD* = 4.838 mm); S1 $M_{distance}$= 19.411 mm (*SD* = 2.734 mm). Thus, mid-LPFC sites were, on average, located further from the surface ($M_{distance\text{-}difference}$ = -2.72 mm, $t(28) = -3.05$, $p = 0.005$) than S1 sites. To approximate the adjusted motor threshold (AdjMT) per subject and TMS site, we used a previously-reported average M1 scalp-cortical distance estimate ($M_{distance}$ = 15.1 mm; *SD* = 2 mm)[19], as precise M1 projections were not available for this study. Following Stokes et al. (2005), we estimated distance-adjusted motor thresholds using the following formula for each TMS site:

$$\text{AdjMT} = \text{MT} + 3 \times (\text{Distance\_TMS\_site} - \text{Distance\_M1})$$

(where AdjMT is the distance-adjusted MT in percentage stimulator output; MT is each participants' motor threshold; Distance_TMS_site is the scalp-to-cortex distance per TMS site, and Distance_M1 is the scalp-to-cortex distance for the M1 site)

We found that distance-adjusted motor thresholds differed by TMS site, ($t(28)$= -3.15, p = 0.004); such that mid-LPFC's was higher (*M* = 59.995 (SD = 16.704)) than S1's (*M* = 51.14 (SD = 11.943)). This indicates that, on average, a higher % of stimulator intensity would be theoretically required to target mid-LPFC sites in our study with the same intensity as S1—although note that there was considerable variability across subjects for both sites.

We next examined whether TMS-site induced changes in the magnitude of mid-LPFC multivariate decoding as well as changes in No-Go accuracy were associated with individual differences in the distance-adjusted TMS intensities as a function of TMS site (Δ AdjMT DLPFC – S1). We found that differences in the distance-adjusted thresholds between TMS sites were unrelated to the TMS-induced reduction in mid-LPFC (vs. S1) decoding accuracy (Δ S1–mid-LPFC Classifier AUC scores), ($r(27)$ = -0.1504, p = 0.44), or to the TMS-induced reduction in No-Go Negative (vs. Positive) task performance (Δ S1–mid-LPFC No-Go Accuracy (Negative – Positive), ($r(27)$ = 0.087, p = 0.65).

Thus, while TMS scalp-to-cortical distances differed by TMS (with larger scalp-to-cortex differences observed in mid-LPFC compared to S1), distance-adjusted motor thresholds were unrelated to the magnitude of the observed TMS-induced neural and behavioral

effects (across subjects). However, note that individualized scalp-to-cortex M1 distances were not available for this study—therefore, our present calculation primarily addresses whether relative distances between our two TMS sites differed, as well as the potential behavioral and neural correlates of individual differences in those distances. To estimate the adjusted MT with maximal precision, given that the cortical depth of the hand area can vary across subjects[19], future studies should obtain individualized scalp-cortical distances in M1.

Table S1. Results of the functional connectivity analysis (PPI) during negative emotional processing as a function of cTBS site (mid-LPFC vs. Control/S1). The regions below showed reduced functional coupling with mid-LPFC during negative emotional processing after cTBS was administered to mid-LPFC (vs. S1); whole-brain cluster-level corrected for multiple comparisons at $Z < -3.1$, $p < 0.05$.

			Coordinates at Z peak			
Region	*Side*	*Size (mm^3)*	*x*	*y*	*z*	*Z peak*
Precuneus	L/R	5864	-4	-52	16	-4.61
Lateral Occipital	L	3816	-52	-66	20	-4.37
Extrastriate Cortex	L/R	1696	10	-84	30	-4.27
Frontal Pole/mPFC	L/R	1384	-24	44	24	-4.28
Lateral Occipital	R	1256	36	-84	20	-4.26
Temporal Pole	L	928	-46	8	-32	-3.97
Superior Parietal Lobule	R	920	22	-52	66	-3.87
Fusiform Gyrus	L	760	-22	-64	-12	-4.00

L = left, R = right, mPFC = medial prefrontal cortex.

Table S2. Spatial overlap of Yeo 7 networks and the FP/mPFC cluster showing functional connectivity (PPI) changes with mid-LPFC sites during negative emotional processing (cTBS to mid-LPFC vs. Control/S1) (FP/mPFC cluster circled in **Figure 3A**; see also **Table S1**)

Yeo network number	Network Name	Number of Overlapping Voxels	% of Overlapping Voxels	% Yeo
1	visual	0	0.000	0.00
2	motor	0	0.000	0.00
3	DA	0	0.000	0.00
4	VA	23	13.295	0.17
5	limbic	0	0.000	0.00
6	FPN	12	6.936	0.06
7	DMN	100	57.803	0.33

DA = dorsal attention, VA = ventral attention, FPN = frontoparietal network, DMN = default mode network.

Table S3. Spatial overlap of Yeo 7 networks and whole-brain functional connectivity (PPI) changes of mid-LPFC sites during negative emotional processing (cTBS to mid-LPFC vs. Control/S1) (whole-brain corrected cluster shown in **Figure 3A**; see also **Table S1**)

Yeo network number	Network Name	Number of Overlapping Voxels	% of Overlapping Voxels	% Yeo
1	visual	722	34.745	3.24
2	motor	1	0.048	0.01
3	DA	146	7.026	0.95
4	VA	23	1.107	0.17
5	limbic	7	0.337	0.06
6	FPN	15	0.722	0.08
7	DMN	968	46.583	3.21

DA = dorsal attention, VA = ventral attention, FPN = frontoparietal network, DMN = default mode network.

References

1. Bender, A. D., Filmer, H. L., Garner, K. G., Naughtin, C. K. & Dux, P. E. On the relationship between response selection and response inhibition: An individual differences approach. *Atten. Percept. Psychophys.* **78**, 2420–2432 (2016).
2. Kertzman, S. *et al.* Go-no-go performance in pathological gamblers. *Psychiatry Res.* **161**, 1–10 (2008).
3. Weafer, J., Baggott, M. J. & de Wit, H. Test-retest reliability of behavioral measures of impulsive choice, impulsive action, and inattention. *Exp. Clin. Psychopharmacol.* **21**, 475–481 (2013).
4. Hedge, C., Powell, G. & Sumner, P. The reliability paradox: Why robust cognitive tasks do not produce reliable individual differences. *Behav. Res. Methods* **50**, 1166–1186 (2018).
5. Enkavi, A. Z. *et al.* Large-scale analysis of test–retest reliabilities of self-regulation measures. *Proceedings of the National Academy of Sciences* **116**, 5472–5477 (2019).
6. Zhuang, Q. *et al.* Segregating domain-general from emotional context-specific inhibitory control systems - ventral striatum and orbitofrontal cortex serve as emotion-cognition integration hubs. *Neuroimage* **238**, 118269 (2021).
7. Bos, D. J. *et al.* Distinct and similar patterns of emotional development in adolescents and young adults. *Dev. Psychobiol.* **62**, 591–599 (2020).
8. Tottenham, N., Hare, T. A. & Casey, B. J. Behavioral assessment of emotion discrimination, emotion regulation, and cognitive control in childhood, adolescence, and adulthood. *Front. Psychol.* **2**, 39 (2011).
9. Somerville, L. H., Hare, T. & Casey, B. J. Frontostriatal maturation predicts cognitive control failure to appetitive cues in adolescents. *J. Cogn. Neurosci.* **23**, 2123–2134 (2011).
10. Hare, T. A. *et al.* Biological substrates of emotional reactivity and regulation in adolescence during an emotional go-nogo task. *Biol. Psychiatry* **63**, 927–934 (2008).
11. Hare, T. A., Tottenham, N., Davidson, M. C., Glover, G. H. & Casey, B. J. Contributions of amygdala and striatal activity in emotion regulation. *Biol. Psychiatry* **57**, 624–632 (2005).
12. Tottenham, N. *et al.* Prolonged institutional rearing is associated with atypically large amygdala volume and difficulties in emotion regulation. *Dev. Sci.* **13**, 46–61 (2010).
13. Schel, M. A. & Crone, E. A. Development of response inhibition in the context of relevant versus irrelevant emotions. *Front. Psychol.* **4**, 383 (2013).
14. Putman, P., van Peer, J., Maimari, I. & van der Werff, S. EEG theta/beta ratio in relation to fear-modulated response-inhibition, attentional control, and affective traits. *Biol. Psychol.* **83**, 73–78 (2010).
15. Zhang, J., Feng, C. & Mai, X. Automatic emotion regulation in response inhibition: The temporal dynamics of emotion counter-regulation during a go/no-go task. *Psychophysiology* **53**, 1909–1917 (2016).
16. Yang, S. *et al.* Emotional content modulates response inhibition and perceptual processing. *Psychophysiology* **51**, 1139–1146 (2014).

17. Morey, R. D. Confidence Intervals from Normalized Data: A correction to Cousineau (2005). *Tutor. Quant. Methods Psychol.* **4**, 61–64 (2008).
18. Yeo, B. T. T. *et al.* The organization of the human cerebral cortex estimated by intrinsic functional connectivity. *J. Neurophysiol.* **106**, 1125–1165 (2011).
19. Stokes, M. G. *et al.* Simple Metric For Scaling Motor Threshold Based on Scalp-Cortex Distance: Application to Studies Using Transcranial Magnetic Stimulation. *J. Neurophysiol.* **94**, 4520–4527 (2005).
