# Supplementary material for: Information-based TMS to mid-lateral prefrontal cortex disrupts action goals during emotional processing
Source: Nat Commun. 2024 May 20;15:4294. doi: 10.1038/s41467-024-48015-8 (PMC11106324; doi:10.1038/s41467-024-48015-8)
Supplement: Supplementary file 3 — Reporting Summary [file 41467_2024_48015_MOESM3_ESM.pdf]

nature portfolio

Corresponding author(s): Regina C Lapate

Last updated by author(s): Mar 23, 2024

Reporting Summary

Nature Portfolio wishes to improve the reproducibility of the work that we publish. This form provides structure for consistency and transparency in reporting. For further information on Nature Portfolio policies, see our Editorial Policies and the Editorial Policy Checklist.

Statistics

For all statistical analyses, confirm that the following items are present in the figure legend, table legend, main text, or Methods section.

n/a	Confirmed	
☐	☒	The exact sample size (*n*) for each experimental group/condition, given as a discrete number and unit of measurement
☐	☒	A statement on whether measurements were taken from distinct samples or whether the same sample was measured repeatedly
☐	☒	The statistical test(s) used AND whether they are one- or two-sided<br>*Only common tests should be described solely by name; describe more complex techniques in the Methods section.*
☐	☒	A description of all covariates tested
☐	☒	A description of any assumptions or corrections, such as tests of normality and adjustment for multiple comparisons
☐	☒	A full description of the statistical parameters including central tendency (e.g. means) or other basic estimates (e.g. regression coefficient) AND variation (e.g. standard deviation) or associated estimates of uncertainty (e.g. confidence intervals)
☐	☒	For null hypothesis testing, the test statistic (e.g. *F*, *t*, *r*) with confidence intervals, effect sizes, degrees of freedom and *P* value noted<br>*Give P values as exact values whenever suitable.*
☒	☐	For Bayesian analysis, information on the choice of priors and Markov chain Monte Carlo settings
☒	☐	For hierarchical and complex designs, identification of the appropriate level for tests and full reporting of outcomes
☐	☒	Estimates of effect sizes (e.g. Cohen's *d*, Pearson's *r*), indicating how they were calculated

Our web collection on statistics for biologists contains articles on many of the points above.

Software and code

Policy information about availability of computer code

Data collection	PsychoPy 2 (v. 1.79.01) run in Python.
Data analysis	R Studio Version 1.0.153. R version 4.0.2. Packages: tidyr_1.1.0; dplyr_1.0.0; emmeans_1.4.8; ggplot2_3.4.0; lme4_1.1-23; lmerTest_3.1-2; lsr_0.5; reshape2_1.4.4; Nilearn (Python v. 2.7) .

For manuscripts utilizing custom algorithms or software that are central to the research but not yet described in published literature, software must be made available to editors and reviewers. We strongly encourage code deposition in a community repository (e.g. GitHub). See the Nature Portfolio guidelines for submitting code & software for further information.

Data

Policy information about availability of data

All manuscripts must include a data availability statement. This statement should provide the following information, where applicable:

- Accession codes, unique identifiers, or web links for publicly available datasets
- A description of any restrictions on data availability
- For clinical datasets or third party data, please ensure that the statement adheres to our policy

Study materials, including emotional face stimuli as well as the behavioral and fMRI data analyzed in this study are available online in OSF at: https://osf.io/3tjsg/. Source data are provided with this paper and available here: https://osf.io/uza3v. For access to the PFC consensus atlas, please email: Dr. Lennart Verhagen at lennart.verhagen@donders.ru.nl.

Research involving human participants, their data, or biological material

Policy information about studies with human participants or human data. See also policy information about sex, gender (identity/presentation), and sexual orientation and race, ethnicity and racism.

Reporting on sex and gender	Thirty-one participants were recruited from Berkeley, CA (n=17 female; based on self report). Data on sex/gender were self-reported. Analysis based on sex or gender were not conducted because we did not have a-priori hypotheses that the effect of DLPFC cTBS would vary based on sex or gender. Given the nature of this study--i.e., multi-session, within subjects design, N=31-- we are not sufficiently powered to examine effects broken down by sex or gender.
Reporting on race, ethnicity, or other socially relevant groupings	N/A
Population characteristics	Thirty one participants (M = 22.5 y old; SD = 3.32; range = 18-29; 17 female)
Recruitment	Participants were recruited from the local community in Berkeley, CA using UC Berkeley email lists as well as posts in the community (Craigslist). We do not anticipate self-selection bias concerns.
Ethics oversight	All procedures were approved by the UC Berkeley Committee for the Protection of Human Subjects.

Note that full information on the approval of the study protocol must also be provided in the manuscript.

Field-specific reporting

Please select the one below that is the best fit for your research. If you are not sure, read the appropriate sections before making your selection.

☐ Life sciences ☒ Behavioural & social sciences ☐ Ecological, evolutionary & environmental sciences

For a reference copy of the document with all sections, see nature.com/documents/nr-reporting-summary-flat.pdf

Behavioural & social sciences study design

All studies must disclose on these points even when the disclosure is negative.

Study description	The data are experimental (within-subjects design) and quantitative.
Research sample	Thirty-one participants were recruited from Berkeley, CA (M = 22.5 y old; SD = 3.32; range = 18-29; 17 female). Sample size was chosen based on prior within-subjects TMS studies of prefrontal function and cognitive control (e.g. Nee et al. 2016 eLife; Ranhnev et al. 2018) (21-24 pps), where we aimed to maximize initial recruitment due to the number of sessions required to complete the study (N=3 fMRI sessions total; 2 TMS+fMRI). Following a baseline fMRI session (N=37) all participants who were invited to participate in the subsequent TMS+fMRI sessions (N=31) returned for all sessions. Eligible participants were healthy, with no self-reported history of psychiatric or neurological disorders, and had normal or corrected-to-normal vision. Written informed consent was obtained from each subject at the University of California, Berkeley. All procedures were approved by the UC Berkeley Committee for the Protection of Human Subjects. Participants were compensated monetarily for their participation.
Sampling strategy	Sampling was random from a convenience sample recruited from the University of California, Berkeley and the local Berkeley community. Sample size was chosen based on prior within-subjects TMS studies of prefrontal function and cognitive control (e.g. Nee et al. 2016 eLife; Shekhar & Rahnev et al. 2018) (21-24 pps), where we aimed to maximize initial recruitment due to the number of sessions required to complete the study (N=3 fMRI sessions total; 2 TMS+fMRI). Following a baseline fMRI session (N=37) all participants who were invited to participate in the subsequent TMS+fMRI sessions (N=31) returned for all sessions. No participants were excluded.
Data collection	Following MRI and TMS safety screening, participants underwent a baseline (no TMS) fMRI session wherein they completed the Affective Go/No-Go task in the MRI scanner. T1-weighted scans were obtained and subsequently used for neuronavigation during the TMS sessions. Participants returned for two additional sessions where offline TMS was administered and followed immediately by completion of the AGNG task inside of the MRI scanner while fMRI data were acquired.<br><br>The two TMS+fMRI sessions took place on two separate days. Continuous theta- burst TMS (cTBS) was delivered to either mid-LPFC (based on the location of multivariate action-goal representations (Go vs. No-Go decoding) identified in the baseline session), or a control site (medial S1). TMS site order was counterbalanced across participants. The two TMS+fMRI sessions were scheduled as closely as possible based on participants' availability and took place on average 5.63 (SD=8.24) days apart. Each TMS+fMRI session began with a metal screening, which was followed by a motor thresholding procedure. Before and after the experiment, participants filled out a mood questionnaire (PANAS Now). At least n=2 researchers were present during TMS administration and data collection (RCL and one or more undergraduate research assistants). The experiment was single blind.<br><br>TMS stimulation protocol<br>TMS was delivered with a Magstim Super Rapid 2 magnetic stimulator (Magstim, Whitland, UK) using a figure-eight double air stimulating coil with a 70mm diameter. Precise TMS targeting on a subject-by-subject basis was achieved using a computerized

	frameless stereotaxic system (Brainsight, Rogue Research) to map the position of the coil and the subject's head in relation to the space of the individual's T1-weighted high- resolution anatomical MRI scan. To temporarily disrupt function of mid-LPFC and Control/S1 sites, we used a continuous TMS protocol (cTBS) consisting of 50Hz trains of 3 TMS pulses repeated every 200 ms continuously over a period of 40 seconds (600 pulses total). This 40-s cTBS protocol has been shown to depress activity in the stimulated brain region for up to 50 min after stimulation31. Throughout the TMS sessions, experimenters actively maintained the TMS coil in a stable position aided by a MagStim coil holder and continuous monitoring of real-time stereotaxic tracking. We delivered cTBS at 80% of active motor threshold, as typically done with this protocol16,31,72. Participant's active motor threshold was defined as the lowest stimulus intensity that elicited at least five twitches and/or sensations in 10 consecutive TMS single pulses delivered to the motor cortex while the subject maintained a voluntary contraction of index and thumb fingers at about 20% of maximum strength. cTBS was delivered with the coil placed tangentially to the scalp, and with the handle pointing posteriorly.
Timing	Baseline sessions were acquired in the Winter/Spring 2018, beginning 3/13/2018 and ending 5/4/2018. TMS+fMRI sessions were acquired subsequently (after baseline sessions were complete), beginning 5/21/2018 and ending 10/19/2021.
Data exclusions	No subjects were excluded from analyses.
Non-participation	No participants dropped out/declined participation.
Randomization	The design is within-subjects--all subjects underwent all experimental conditions. Assignment to "LPFC site first" vs. "S1 site first" was counterbalanced across subjects.

Reporting for specific materials, systems and methods

We require information from authors about some types of materials, experimental systems and methods used in many studies. Here, indicate whether each material, system or method listed is relevant to your study. If you are not sure if a list item applies to your research, read the appropriate section before selecting a response.

Materials & experimental systems

n/a	Involved in the study	
☒	☐	Antibodies
☒	☐	Eukaryotic cell lines
☒	☐	Palaeontology and archaeology
☒	☐	Animals and other organisms
☒	☐	Clinical data
☒	☐	Dual use research of concern
☒	☐	Plants

Methods

n/a	Involved in the study	
☒	☐	ChIP-seq
☒	☐	Flow cytometry
☐	☒	MRI-based neuroimaging

Magnetic resonance imaging

Experimental design

Design type	Event related.
Design specifications	Each Affective Go/No-Go (AGNG) fMRI session totaled 80 trials/run (n=480 trials total across the task, divided into 6 runs) and took ~40 minutes to complete.
Behavioral performance measures	Accuracy and Reaction Times were recorded throughout the task. Means and SEM were computed.

Acquisition

Imaging type(s)	Functional and structural
Field strength	3T
Sequence & imaging parameters	Neuroimaging data were acquired in the UC Berkeley Henry H. Wheeler, Jr. Brain Imaging Center with a Siemens TIM/Trio 3T MRI scanner with a 32-channel RF head coil. Whole-brain Blood Oxygen Level-Dependent (BOLD) functional Magnetic Resonance Imaging (fMRI) data were obtained using a T2*-weighted 2x accelerated multiband echo- planar imaging (EPI) sequence (52 axial slices, 2.5 mm3 isotropic voxels; 84 x 84 matrix, TR= 2000 ms; TE= 30.2 ms; flip angle = 80°; 222 image volumes per run). High-resolution T1-weighted MPRAGE gradient-echo sequence images were collected at the end of the session for spatial normalization and TMS neuronavigation (176 x 256 x 256 matrix of 1 mm3 isotropic voxels; TR = 2300 ms; TE = 2.98 ms; flip angle = 9°).
Area of acquisition	whole brain

Diffusion MRI ☐ Used ☒ Not used

Preprocessing

Preprocessing software

Functional neuroimaging data were processed using FEAT; FSL version 6.0.160,61. Preprocessing steps included removal of the first four functional volumes, high-pass filtering (90 s cutoff), FILM correction for autocorrelation in the BOLD signal, slice-time correction, and motion correction using MCFLIRT. Standard and extended motion parameters (i.e., their temporal derivatives and their squares) and a confound matrix containing points of framewise displacement greater than 0.5mm were used as regressors of non-interest in the analyses to control for movement-confounded activation. Data were smoothed with using a 3mm full width at half maximum (FWHM) Gaussian filter.

Normalization

Functional images were co-registered to participant's T1-weighted anatomical image using a linear rigid body (6-DOF) transform while maintaining native functional resolution (2.5 mm3 isotropic).

Normalization template

Analyses were conducted on participants' structural space (see normalization above).

Noise and artifact removal

Using FSL, FILM correction for autocorrelation in the BOLD signal was applied, and motion correction was done using MCFLIRT. Standard and extended motion parameters (i.e., their temporal derivatives and their squares) and a confound matrix containing points of framewise displacement greater than 0.5mm were used as regressors of non-interest in the analyses to control for movement-confounded activation.

Parameter estimates extracted from each ROI were regularized with multivariate noise normalization. To do so, we obtained an estimate of the noise covariance from the residuals of the general linear models from each ROI. This matrix was then regularized using the optimal shrinkage parameter, inverted, and multiplied by the vector of betas for each trial. This approach aims to removes nuisance correlations between voxels that arise due to physiological and instrument noise.

Volume censoring

No volume censoring was done.

Statistical modeling & inference

Model type and settings

MVPA:
We obtained trial-wise BOLD activation parameters estimates using the Least- Squares All (LS-A) GLM approach and FEAT modeling in FSL61. Single trials were modeled using a canonical Double γ hemodynamic response function.

To test whether cTBS to mid-LPFC altered action goal representations, we examined classifier performance of Go vs. No-Go classes from multivoxel neural activity patterns extracted from individualized mid-LPFC sites during the baseline session (no TMS), as well as following cTBS to mid-LPFC and Control/S1 sites.

We used a linear classifier to examine the decodability of action goal (Go vs. No- Go) from mid-LPFC multivoxel neural activity patterns implemented with Nilearn. For each subject and ROI, we used a multivariate logistic regression model (l2 penalty; C=1) to iteratively train the classifier on z-scored data. We assessed classifier performance using a leave-one-run-out cross validation scheme. Classification performance was evaluated using the area under the curve (AUC) metric (i.e., where 0.5 is chance performance). For all multivariate decoding analyses, we used Nilearn's parameter (class_weight='balanced') to automatically adjust weights according to class frequencies in the input data (important for the classification of Go vs. No-Go classes). To examine whether classifier performance differed from chance, we combined run-wise classifier AUCs (−0.5) across subjects using a mixed-model approach and tested whether the intercept differed significantly from 0 (subject and run were entered as random factors). This constituted the primary analysis reported in the manuscript. To test whether classifier performance differed by TMS session, we entered TMS condition as a fixed factor in the same mixed model predicting classifier accuracy (AUC; subject and run were entered as random factors).

To additionally validate our results using a non-parametric method (i.e., robust to violations of normality assumptions), we derived a null distribution of classifier performance by shuffling labels (n=500 permutations). (Supplementary Results)

Functional Connectivity
To examine whether cTBS to LPFC resulted in circuit- level changes in mid-LPFC connectivity, we used psychophysiological interaction analysis (PPI). To do so, we first extracted the mean time series from individualized mid-LPFC sites. We then ran a separate FEAT analysis for each run that included emotional valence regressors, the demeaned timecourse of participants' mid-LPFC seed as well as the interaction between this timecourse and regressors for negative and positive emotional stimuli. At the group level, we examined the result of these whole-brain analyses cluster-corrected for multiple comparison at $Z = 3.1$, $p < 0.05$.

Effect(s) tested

Classifier performance (decodability) of Go vs. No-Go (and Emotional Valence) from mid-LPFC as a function of TMS condition. Functional Connectivity changes (Psychophysiological Interaction analysis/PPI) of mid-LPFC a function of TMS condition.

Specify type of analysis: ☐ Whole brain ☒ ROI-based ☐ Both

Anatomical location(s)	Anatomical ROIs. Prefrontal ROIs used for anatomical inference of functional results—frontal pole (FPl & FPm) and mid-LPFC (BA46 & 9-46)— were obtained from the Oxford PFC consensus atlas, thresholded at 25%, and registered to participants' native surface space using Freesurfer65. As described previously (Lapate et al. J Neuro 2022), vertex coordinates were transformed into the native (volumetric) space, and ROI masks in volumetric space were constructed by projecting half the distance of the cortical thickness at each vertex, requiring that a functional voxel be filled at least 50% by the label, and labeling the intersected voxels.<br><br>Functional ROIs. A 5mm3 sphere surrounding subject-specific mid-LPFC coordinates (see TMS sites) was created for extraction of multivariate action-goal (Go vs. No-Go) decoding as a function of TMS site (no TMS, mid-LPFC and Control/S1).
Statistic type for inference<br>(See Eklund et al. 2016)	For PPI whole-brain functional connectivity analysis of LPFC targets as a function of TMS condition, analyses were cluster-corrected for multiple comparison at $Z = 3.1$, $p < 0.05$.
Correction	Gaussian random field theory (cluster thresholding $Z = 3.1$, $p < 0.05$); FWER.

Models & analysis

n/a	Involved in the study
☐	☒ Functional and/or effective connectivity
☒	☐ Graph analysis
☐	☒ Multivariate modeling or predictive analysis

Functional and/or effective connectivity	To examine whether cTBS to LPFC resulted in circuit- level changes in mid-LPFC connectivity, we used psychophysiological interaction analysis (PPI). To do so, we first extracted the mean time series from individualized mid-LPFC sites. We then ran a separate FEAT analysis for each run that included emotional valence regressors, the demeaned timecourse of participants' mid-LPFC seed as well as the interaction between this timecourse and regressors for negative and positive emotional stimuli. At the group level, we examined the result of these whole-brain analyses cluster-corrected for multiple comparison at $Z = 3.1$, $p < 0.05$.
Multivariate modeling and predictive analysis	We used a linear classifier to examine the decodability of action goal (Go vs. No- Go) from mid-LPFC multivoxel neural activity patterns implemented with Nilearn. For each subject and ROI, we used a multivariate logistic regression model (l2 penalty; C=1) to iteratively train the classifier on z-scored data. We assessed classifier performance using a leave-one-run-out cross validation scheme. Classification performance was evaluated using the area under the curve (AUC) metric (i.e., where 0.5 is chance performance). For all multivariate decoding analyses, we used Nilearn's parameter (class_weight='balanced') to automatically adjust weights according to class frequencies in the input data.
